# Alternating ibuprofen and acetaminophen in the treatment of febrile children: a pilot study [ISRCTN30487061]

**DOI:** 10.1186/1741-7015-4-4

**Published:** 2006-03-04

**Authors:** Mona M Nabulsi, Hala Tamim, Ziyad Mahfoud, Mohammad Itani, Ramzi Sabra, Fadi Chamseddine, Mohammad Mikati

**Affiliations:** 1 Department of Pediatrics, American University of Beirut Medical Center, Beirut, Lebanon; 2 Department of Epidemiology and Population Health, Faculty of Health Sciences, American University of Beirut, Beirut, Lebanon; 3 Department of Pediatrics, Najjar Hospital, Beirut, Lebanon; 4 Department of Pharmacology and Therapeutics, Faculty of Medicine, American University of Beirut, Beirut, Lebanon

## Abstract

**Background:**

Alternating ibuprofen and acetaminophen for the treatment of febrile children is a prevalent practice among physicians and parents, despite the lack of evidence on effectiveness or safety. This randomized, double-blind and placebo-controlled clinical trial aims at comparing the antipyretic effectiveness and safety of a single administration of alternating ibuprofen and acetaminophen doses to that of ibuprofen mono-therapy in febrile children.

**Methods:**

Seventy febrile children were randomly allocated to receive either a single oral dose of 10 mg/kg ibuprofen and 15 mg/kg oral acetaminophen after 4 hours, or a similar dose of ibuprofen and placebo at 4 hours. Rectal temperature was measured at baseline, 4, 5, 6, 7 and 8 hours later. Endpoints included proportions of afebrile children at 6, 7 and 8 hours, maximum decline in temperature, time to recurrence of fever, and change in temperature from baseline at each time point. Intent-to-treat analysis was planned with statistical significance set at P < 0.05.

**Results:**

A higher proportion of subjects in the intervention group (83.3%) became afebrile at 6 hours than in the control group (57.6%); P = 0.018. This difference was accentuated at 7 and 8 hours (P < 0.001) with a significantly longer time to recurrence of fever in the intervention group (mean ± SD of 7.4 ± 1.3 versus 5.7 ± 2.2 hours), P < 0.001. Odds ratios (95%CI) for defervescence were 5.6 (1.3; 23.8), 19.5 (3.5; 108.9) and 15.3 (3.4; 68.3) at 6, 7 and 8 hours respectively. Two-way ANOVA with repeated measures over time revealed a significantly larger decline in temperature in the intervention group at times 7 (P = 0.026) and 8 (P = 0.002) hours.

**Conclusion:**

A single dose of alternating ibuprofen and acetaminophen appears to be a superior antipyretic regimen than ibuprofen mono-therapy. Further studies are needed to confirm these findings.

## Background

Fever, a beneficial immune host response [1], is often a cause of significant anxiety among parents of febrile children. Fever phobia [2], which refers to unproven concerns about fever causing serious harm, is widely prevalent among caregivers [2,3], and may lead to abuse of antipyretics with subsequent risk of toxicity. Acetaminophen and ibuprofen are the most commonly used antipyretics in pediatrics. These two drugs have well-established efficacy and safety profiles, when used in appropriate dosages [4,5]. Recently, however, we have observed the emergence and increasing popularity of a new antipyretic regimen in Lebanon that combines acetaminophen and ibuprofen in alternating doses. This practice has also been reported from America and Europe [6-9]. The new trend of combining ibuprofen and acetaminophen for treating fever has not been studied in clinical trials for efficacy or safety. In fact, several investigators have raised concerns that such combinations may result in significant adverse effects on the liver or the kidney [7-9]. Specifically, the risk of renal toxicity may increase when both drugs are used together, since ibuprofen reduces glutathione and, in the presence of increased concentrations of acetaminophen in the kidney and reduced glutathione, can lead to renal tubular necrosis [7-9].

In view of the present popularity of the combined alternating ibuprofen and acetaminophen treatment of febrile children, the potential risks associated with it, and the absence of scientific evidence on its safety or superior effectiveness to mono-therapy, we elected to conduct this randomized controlled trial that aims at comparing the effectiveness and safety of a single administration of combined alternating ibuprofen and acetaminophen doses to that of ibuprofen mono-therapy. We chose to investigate the antipyretic effectiveness and safety of a single dose of each regimen, rather than multiple doses, because we believed it would be unethical to expose our subjects to possible harm from multiple doses prior to having scientific evidence of single-dose effectiveness.

## Methods

### Setting

This study was conducted between November 2002 and April 2005, in the paediatric inpatient services of two hospitals in Beirut: the American University of Beirut Medical Centre (AUBMC), which is a tertiary care facility; and Najjar Hospital, a secondary care facility. The study was approved by the Institutional Review Board and the Ethics Committee of the American University of Beirut, as well as the Board of Najjar Hospital.

### Subjects

Eligible subjects were febrile inpatients aged between 6 months and 14 years, whose rectal temperature was ≥ 38.8°C. Exclusion criteria included any of the following conditions: vomiting, any medical or surgical condition that precluded oral drug administration, acute or chronic hepatic disease, malabsorption syndromes, acute or chronic renal disease with the exception of urinary tract infection, chronic metabolic disease, bleeding disorders, asthma, chronic neurological disease that may affect central thermoregulation, cancer, immune suppression, sepsis, critical medical status, or known allergy to acetaminophen or ibuprofen. Children with concurrent or previous intake of antibiotics were not excluded if still febrile at the time of interview. All antipyretics were stopped for 8 hours prior to the initiation of the study.

### Study design

This was a randomized, double-blind and placebo-controlled clinical trial in which subjects were randomly allocated into one of two treatment groups: an intervention group where a single oral dose of 10 mg/kg ibuprofen was administered at baseline followed by a single oral dose of 15 mg/kg acetaminophen four hours later; and a control group where a similar dose of ibuprofen was administered initially, followed by placebo four hours later. The allocation sequence was generated by one of the co-investigators (HT) who was not involved in subject recruitment, drug administration or outcome assessment. The administration of acetaminophen or placebo four hours after baseline was chosen to coincide with the expected time of maximum antipyresis of ibuprofen, after which there is gradual waning of this effect. Hence, we anticipated that the antipyretic action of acetaminophen may result in further control of the temperature between 4 and 8 hours from baseline.

### Study procedure

After obtaining the approval of the treating physician, the parent(s) of the eligible child was/were approached for interview and enrolment. During the interview, a trained research assistant who was responsible for subject enrolment administered a structured questionnaire designed to collect information on the following variables: gender; date of birth; maternal and paternal levels of education; maternal and paternal jobs; fever duration; diagnosis; previous or concurrent antibiotic intake; previous antipyretic intake; previous use of ibuprofen and acetaminophen in alternating schedule; the person who recommended the alternating antipyretic regimen; parental rating of the alternating antipyretic regimen effectiveness. The purpose and procedure of the trial were fully explained to the family, and written parental consent was obtained, together with the assent of the subject if older than ten years. Children enrolled in the study were then assigned a random number by the hospital pharmacist according to a computer-generated random-number list, which was kept with the pharmacist until the end of the study. The pharmacist who prepared all study medications was thus un-blinded to the treatment allocation of subjects, while subjects, parents, research assistant, nurses responsible for drug administration and outcome assessment, treating physicians, data analyst (co-investigator ZM) and remaining investigators were all blinded to the patients' assignment.

Baseline rectal temperature was recorded using a portable thermistor with single-use disposable probe covers (Sure Temp 679, Welch Allyn). The same thermometer was used for the whole duration of the study. Rectal temperatures were subsequently recorded by the nurse in charge of the subject's care at 4, 5, 6, 7 and 8 hours from baseline.

### Study medications

The drugs used in this study, ibuprofen, acetaminophen and its placebo, were supplied by Julphar (Gulf Pharmaceutical Industries, United Arab Emirates). The oral ibuprofen used was a 100 mg/5 ml suspension (Profinal, Julphar), while oral acetaminophen was a 250 mg /5 ml suspension (Adol, Julphar) and its placebo was a suspension with similar colour and exipient to Adol.

### Statistical analyses

The primary outcome of the study was the proportion of children with normal body temperature at 6 hours. Normal temperature was defined as a rectal measurement ranging between 36.5°C and 37.9°C. We hypothesized that 50% of febrile subjects who receive ibuprofen and placebo will drop their rectal temperature to <38.0°C at 6 hours, and that 80% of subjects in the combination antipyretic group will become afebrile at 6 hours. To detect this 30% difference in the proportions of afebrile subjects, with α of 0.05, β of 20% and a 2-tailed test for the difference in proportions, a sample size of 90 subjects is needed: 45 in each group. Additional outcomes included: proportions of afebrile children in each group at 7 and 8 hours from baseline; maximum decline in temperature during the study period; time to recurrence of fever; the mean temperature changes from baseline at t = 4, 5, 6, 7 and 8 hours; the proportion of patients in each group with any adverse effect that may be related to either drug such as hypothermia, chilliness or gastrointestinal bleeding.

Statistical analyses were performed using SPSS, version 12.0. We investigated the association between categorical variables and treatment groups with Pearson's Chi Square test, and the relationship between continuous variables and treatment groups with Student's t-test. Data were also analyzed using two-way ANOVA with repeated measures over time. Error terms were modeled as having a covariance structure of the first-order autoregressive type. In order to eliminate bias that could be caused by baseline temperature, the variable chosen for analysis was the change in temperature, from baseline, at times t (t = 4, 5, 6, 7, 8). Additional testing with logistic regression was performed to investigate the association between the proportion of afebrile subjects at 6 hours as the dependent variable and the treatment group as the independent variable, adjusting for temperature at 4 hours, previous antibiotic intake and previous antipyretic intake. Similar regression analyses were used to test the associations between the same predictors and the proportions of afebrile subjects at 7 and 8 hours as dependent variables. Odds ratios with 95% confidence intervals were calculated. Intent-to-treat analysis was planned with statistical significance set at P < 0.05.

## Results

### Baseline characteristics

Between November 2002 and April 2005, 143 parents were approached for interview and questionnaire administration. Of these, 55 (38.5%) admitted having used ibuprofen and acetaminophen on alternating basis, of whom 84.3% initiated this practice following a physician's advice while in 13.7% it was self-initiated. The practice was rated as being very effective in treating high fever by 71.7% of parents who used the regimen.

**Figure 1:**
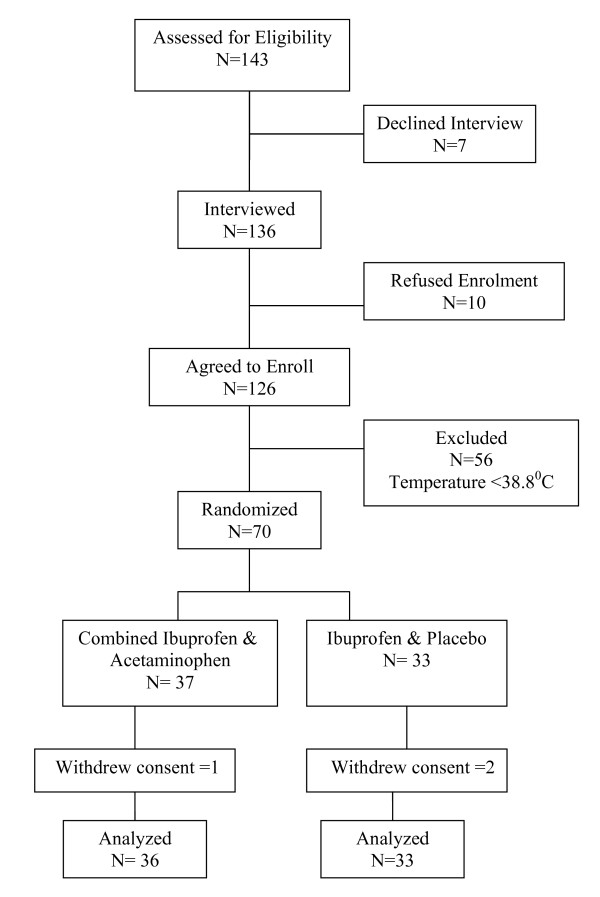
Flow diagram of the subjects' progress through the study.

Figure 1 summarizes the progress of these subjects through the study, which was terminated after two and a half years owing to a very slow recruitment rate. The final sample size enrolled was 70 subjects, randomized either to the ibuprofen and acetaminophen combination treatment group (37) or the ibuprofen and placebo group (33). One patient from the intervention group withdrew consent at 4 hours and could not be included in the final analysis because the parents refused temperature monitoring. Two patients from the control group withdrew consent at 6 hours; they were kept for the intent to treat analysis at 6 hours, but no temperature recordings were obtained at 7 and 8 hours.

Except for gender and the type of father's job, baseline characteristics were not significantly different between those who were enrolled in the study and those who refused. There was a higher proportion of males among enrolled subjects (64.3%) as compared to non-enrolled (47.9%), P = 0.049. In addition, 83.3% of fathers with a manual job consented to the study, in contrast to fathers with administrative or academic jobs (46.0%), P = 0.045.

As for the randomized subjects (70), the overall mean (SD) age was 3.7 (3.1) years, with an age range of 6 months-12.8 years. The mean (SD) duration of fever was 4.7 (4.1) days, with a range of 1.0–30.0 days. One patient had a prolonged fever of 30 days that was later diagnosed as due to tuberculosis. Those enrolled included 45 (64.3%) males and 45 (67.2%) subjects receiving antibiotic treatment. The characteristics of the two groups are shown in Table 1. There were no significant differences between the two treatment groups with respect to gender, age, underlying basic disease causing fever, duration of fever, previous antipyretic use, concurrent antibiotic administration, mean baseline temperature (39.3 ± 0.5°C) or mean temperature at 4 hours (37.5 ± 0.7 °C), at the time of acetaminophen/placebo administration (Table 1). In addition, there were no differences in the treatment allocation of the patients recruited from AUBMC (32) and those recruited from Najjar Hospital (38).

### Primary and secondary outcomes

A significantly larger number of subjects in the intervention group (30/36; 83.3%) achieved a normal body temperature at 6 hours than in the control group (19/33; 57.6%), P = 0.018. This difference persisted at 7 and 8 hours with significantly higher proportions of afebrile subjects in the combined antipyretic group (31/36; 86.1% versus 14/31; 45.2% at 7 hours, and 29/36; 80.6% versus 11/31; 35.5% at 8 hours; P < 0.001 at both times) (Table 2). In univariate logistic regression testing, temperature at 4 hours was found to be a significant predictor of the antipyretic response at 6, 7, and 8 hours (P ≤ 0.001), whereas previous antibiotic or antipyretic intakes were not. The logistic regression model revealed that subjects in the intervention group were significantly more likely than those in the control group to become afebrile at 6, 7 and 8 hours: OR (95% CI) of 5.6 (1.3; 23.8) at 6 hours; 19.5 (3.5; 108.9) at 7 hours; and 15.3 (3.4; 68.3) at 8 hours,.

**Figure 2:**
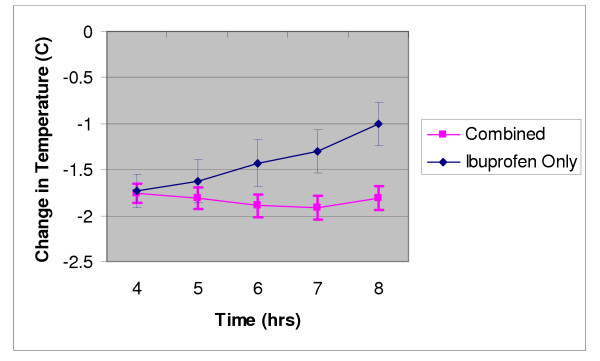
Mean (SE) change in temperature, from baseline, over time in the two treatment groups.

The two groups had similar maximum decline in temperature (mean ± SD of 2.2 ± 0.7°C in the intervention group versus 2.1 ± 1.2°C in the control group; P = 0.8). However, the combined antipyretic group had a significantly longer duration of antipyresis than the control group, with the mean (SD) times to recurrence of fever being 7.4 (1.3) hours versus 5.7 (2.3) hours, respectively; P < 0.001 (Table 2). Two-way ANOVA with repeated measures over time revealed a significant interaction between time and treatment group (P = 0.011), hence the change in temperature followed different trends in the two treatment groups. This is well demonstrated when the change in temperature is plotted against time, as shown in Figure 2. Owing to this significant interaction, comparison of the two groups at each time period was performed with Bonferroni adjustments revealing significant differences, with the intervention group showing larger declines in temperature than the control group at times 7 and 8 hours (P = 0.026 and 0.002, respectively).

As for the side effects of medications, low body temperature (defined as a rectal temperature below 36.5°C) occurred in 11 (15.9%) of the subjects: 5 (13.9%) in the combined antipyretic group and 6 (18.2%) in the control group (P = 0.6). The temperature range of these episodes was between 35.0°C and 36.2°C. No serious adverse reactions were observed in these subjects. In addition, none of the subjects developed any symptom or sign suggestive of gastrointestinal, hepatic or renal toxicity.

## Discussion

Alternating antipyretics is becoming a popular practice among physicians caring for febrile children. Mayoral et al. [6] reported that 50% of physicians surveyed in the United States advised parents to alternate ibuprofen with acetaminophen, despite the absence of any scientific evidence to support the effectiveness or safety of this practice. Younger physicians are more likely to alternate antipyretics, reflecting their anxiety about parental fever phobia [6,9]. Alternating antipyretics is not unique to physicians in the United States but has been reported from other countries as well [9]. It is being widely practiced in our country, as evident from the fact that more than one third of parents interviewed in this study had alternated acetaminophen and ibuprofen previously. This practice was based on physician's advice in 84.3% and was self-initiated in 13.7% of our families, emphasizing the popularity of this regimen. However, combined antipyretic treatment can be dangerous and may precipitate hepatic or renal toxicities due to glutathione pathway impairment, especially in the sick, dehydrated or fasting child [6-8,10-14]. Dosing errors due to confusing dosing schedules may increase the risk of overdose of one or both drugs, with resultant grave consequences.

Our results suggest that a single administration of alternating ibuprofen and acetaminophen doses is a more effective antipyretic regimen than ibuprofen and placebo. A significantly higher proportion of subjects in the combined regimen became afebrile compared to those treated with ibuprofen alone. This antipyretic effect was maintained for an additional two hours in the intervention group, as evidenced by the significantly higher proportions of afebrile subjects at 7 and 8 hours from baseline. Subjects in the intervention group had a longer duration of antipyresis that persisted throughout the study. In addition, they had larger reductions in their body temperatures at 7 and 8 hours from baseline.

### Strengths and limitations

This study is the first clinical trial to investigate whether the combined alternating antipyretic regimen is more effective than ibuprofen mono-therapy. Although our results suggest the superiority of the combined alternating regimen, our findings need to be confirmed in larger trials, since we were forced to stop the trial before achieving our calculated sample size. This was because of obstacles faced during recruitment that were primarily related to parental anxiety regarding children's participation in research, and to physicians' reluctance to permit enrolment of their patients in a clinical trial. Such obstacles have been encountered by many investigators and have been previously reported in the pediatric research literature [15]. Based on the available sample size, and the difference between proportions of afebrile subjects obtained in our results, we recalculated the actual power of the study and found it to be 66% at 6 hours, 92% at 7 hours and 95% at 8 hours, with α = 0.05.

Subjects included in this study were inpatients who are presumed to be sicker than outpatients; hence the validity of generalizing the findings to outpatients may be in question. However, both ibuprofen and acetaminophen are used in similar dosages and at similar intervals in inpatients as well as outpatients, irrespective of the severity of the primary disease. In addition, the antipyretic response associated with either drug does not correlate with the disease being viral or bacterial. Therefore we believe that the findings of this study may apply to hospitalized and outpatient febrile children alike.

Since the outcome of interest in this study was "effectiveness" rather than "efficacy", we did not exclude subjects receiving antibiotics from enrolment, or subjects with prior intake of antipyretics. However, antipyretics were stopped for 8 hours prior to enrolment, the time at which a febrile subject may receive antipyretic treatment in "real clinical life". It may be argued that the antipyretic effects of the drugs investigated are confounded by antibiotic administration and previous antipyretic intake. However, since this was a randomized clinical trial, we anticipated that the randomization process would dilute these effects by distributing the subjects equally among the two treatment groups. Indeed, the proportions of subjects receiving antibiotics and those with prior antipyretic intake were not significantly different between the two groups, suggesting adequate randomization. In addition, and since the desired sample size was not achieved, we adjusted for antibiotic and antipyretic intakes in the logistic regression model, which revealed both variables to be insignificant predictors of the antipyretic response at 6, 7, and 8 hours.

### Implications for practice

Despite the effectiveness of the combined antipyretic regimen shown in this study, we emphasize that our findings should not be used as a justification for advising this practice. The duration of our study was a short 8 hour interval, during which a single dose of ibuprofen and acetaminophen was administered. Although no renal, hepatic or gastrointestinal adverse effects were observed, no definite conclusions on the safety of the combined antipyretic treatment can be made before larger multi-dose clinical trials are conducted demonstrating its safety. In addition, the antipyretic advantage of the combined antipyretics may be attenuated with multiple dosing of ibuprofen and acetaminophen, and become comparable to repeated ibuprofen mono-therapy. A similar situation has been reported by Walson et al. [16], where multi-dose treatments with 2.5 mg/kg and 5 mg/kg ibuprofen for 24–48 hours resulted in equivalent antipyresis to 10 mg/kg ibuprofen or 15 mg/kg acetaminophen, after the second dose and continuing to 24–48 hours later.

## Conclusion

A single administration of alternating ibuprofen and acetaminophen doses to febrile children appears to be a more effective antipyretic than ibuprofen alone. It is our position, however, that combined treatment should not be used in clinical practice before larger clinical trials confirm the safety and effectiveness of this regimen.

## Competing interests

The author(s) declare that they have no competing interests.

## Authors' contributions

MMN prepared grant submission in relation to this study, contributed to the design, data acquisition, analysis and interpretation, drafting, revision and final approval of the manuscript. HT contributed to the design, data analysis and interpretation, drafting, revision and final approval of the manuscript. ZM contributed to statistical analysis, revision and final approval of the manuscript. MI contributed to data acquisition, analysis, drafting and final approval of the manuscript. RS participated in grant submission, design, revision and final approval of the manuscript. FC contributed substantially to data acquisition, drafting and final approval. MM participated in grant submission, drafting, revision and final approval of the manuscript.

## Pre-publication history

The pre-publication history for this paper can be accessed here:



## Supplementary Material

Additional File 1 CONSORT checklist: BMC Consort.doc Click here for file

## Figures and Tables

**Table 1:** Subjects' baseline characteristics.

	**Total (N = 70)**	**Combined ibuprofen & acetaminophen (N = 37)**	**Ibuprofen (N = 33)**	**P value**
**Male gender N (%)**	45 (64.3)	26 (70.3)	19 (57.6)	0.3
**Age (years)**				
Mean (SD)	3.7 (3.1)	3.7 (3.3)	3.6 (2.9)	0.9
Range	0.5–12.8			
**Illness duration (Days)**				
Mean (SD)	4.7 (4.1)	4.6 (4.9)	4.7 (2.9)	0.9
Range	1–30	1–30	1–14	
**Fever aetiology N (%)**				
Viral	44 (62.9)	26 (70.3)	18 (54.5)	
Bacterial	19 (27.1)	8(21.6)	11 (33.3)	0.4
Other	7 (10.0)	3 (8.1)	4 (12.1)	
**Hospital N (%)**				
AUBMC	32 (45.7)	16 (43.2)	16 (48.5)	0.7
Najjar	38 (54.3)	21 (56.8)	17 (51.5)	
**Previous antipyretic N (%)**	68 (98.6)	37 (100)	31 (96.9)	0.3
**Antibiotic intake N (%)**	45 (67.2)	22 (59.5)	23 (76.7)	0.1
**Baseline temperature (°C)**				
Mean (SD)	39.3 (0.5)	39.3 (0.5)	39.4 (0.6)	0.3
**Temperature at 4 hours (°C)**				
Mean (SD)	37.5 (0.7)	37.5 (0.7)	37.7 (0.9)	0.3

**Table 2:** Comparison of primary and secondary outcomes in both treatment groups.

	**Combined Ibuprofen & acetaminophen N = 36**	**Ibuprofen N = 33**	**P value**
**Afebrile at 6 hours N (%)**	30 (83.3)	19 (57.6)	0.018
**Afebrile at 7 hours N (%)**	31 (86.1)	14 (45.2)	<0.001
**Afebrile at 8 hours N (%)**	29 (80.6)	11 (35.5)	<0.001
**Maximum temperature decline Mean (SD)**	2.2 (0.7)	2.1 (1.2)	0.793
